# Genomic characterization of two duck-origin picornaviruses with seven putative 2A peptides

**DOI:** 10.3389/fvets.2026.1753959

**Published:** 2026-04-23

**Authors:** Yuehua Gao, Tong Zhu, Yifei Jiang, Fengjuan Tian, Yufeng Li, Wenli Liu, Shan Xu, Yigang Tong, Zhuoming Qin, Feng Hu

**Affiliations:** 1 Shandong Provincial Key Laboratory of Livestock and Poultry Breeding, Poultry Institute, Shandong Academy of Agricultural Sciences, Jinan, China; 2 BAICSM, State Key Laboratory of Green Biomanufacturing, College of Life Science and Technology, Beijing University of Chemical Technology, Beijing, China; 3 Medical Center of Hematology, Xinqiao Hospital of Army Medical University, Chongqing, China; 4 Institute of Analysis and Testing, Beijing Academy of Science and Technology (Beijing Center for Physical and Chemical Analysis), Beijing, China

**Keywords:** genomic characterization, homology analysis, metagenomic analysis, picornavirus, virus taxonomy

## Abstract

**Introduction:**

The *Picornaviridae* family is a large group of viruses comprising 68 genera. Duck-origin picornaviruses are categorized into four genera, however, the taxonomic status of some recently identified strains remains to be determined.

**Methods:**

In this study, two virus strains isolated from breeding ducks experiencing reduced egg production were identified and characterized through viral metagenomic analysis.

**Results:**

Two viral strains (NC0246 and PX0394) exhibiting the typical picornavirus-like genomic structure were identified and characterized. Notably, both strains exhibit extended 2A sequences that each possesses seven distinct 2A polypeptides considered rare in *Picornaviridae* family. Specifically, NC0246 exhibits a deletion of 73 amino acids (aa) in the region corresponding to 2A4-2A5 when compared to PX0394 indicating the genetic diversity of picornaviruses. Homology analysis revealed that the P1 region of NC0246 was most closely related to duck aalivirus A1, with aa identity of 37.37%. Conversely, the P1 region of PX0394 was most closely related to duck egg-reducing syndrome virus (DERSV), with aa identity of 64.44%. Furthermore, the 2C and 3D proteins of NC0246 and PX0394 was most closely related to DERSV. Phylogenetic analyses indicate that NC0246 and PX0394 form a sister clade to DERSV and duck aalivirus A1 and display marked heterogeneity in the P1 protein. While NC0246 and PX0394 branch nearest to DERSV and duck aalivirus A1, duck hepatitis A virus types 1 and 3, sharing secondary homology, occupy a separate lineage.

**Conclusion:**

Two picornaviruses were identified and characterized from breeding ducks that exhibited decreased egg production. Through genomic structure and homology analysis, these viruses were most closely related to DERSV and duck aalivirus A1. NC0246, PX0394, and the previously reported DERSV show a close evolutionary relationship with the genus *Aalivirus* based on genomic and phylogenetic analyses, suggesting a potential affiliation with this genus.

## Introduction

The *Picornaviridae* family is a large group of viruses comprising five subfamilies: *Caphthovirinae*, *Ensavirinae*, *Heptrevirinae*, *Kodimesavirinae*, and *Paavivirinae*, and three distinct clusters: *Harkavirus*, *Ampivirus*, and unassigned picornaviruses (1). This family encompasses 159 species, which are further categorized into 68 genera (as of March 2024, according to https://www.picornaviridae.com/index.html) (2). The viruses within this family are known to infect a diverse range of hosts, including mammals, birds, reptiles, amphibians, and fish. Notably, avian-related viruses are distributed across at least 21 genera (*Aalivirus*, *Anativirus*, *Avihepatovirus*, *Avisivirus*, *Crahelivirus*, *Gallivirus*, *Gruhelivirus*, *Grusopivirus*, *Harkavirus*, *Kobuvirus*, *Kunsagivirus*, *Ludopivirus*, *Megrivirus*, *Orivirus*, *Oscivirus*, *Parechovirus*, *Passerivirus*, *Poecivirus*, *Pygoscepivirus*, *Sicinivirus*, and *Tremovirus*). Specifically, picornaviruses originating from ducks are classified into four genera: *Aalivirus*, *Anativirus*, *Avihepatovirus*, and *Megrivirus* (3–5). In a previous study, Wang et al. identified a strain of picornavirus closely related to duck hepatitis A virus (DHAV) in ducks, which was designated as GL/12. This strain is characterized by the presence of six 2A structures. Through genomic characterization and phylogenetic analysis, this virus was recognized as the founding member of a novel genus, *Aalivirus* (3). Su et al. (6) reported that the duck egg-reducing syndrome virus (DERSV), which is responsible for decreased egg production in ducks, contains seven 2A fragments. Furthermore, the P1, 2C, 3C, and 3D proteins of DERSV exhibit similarity levels of 64, 76.8, 77.5, and 70.7%, respectively, with the wild duck avihepatovirus-like virus. Consequently, these proteins are considered to still classify DERSV within the *Avihepatovirus* genus.

In this study, we identified and characterized two viral strains exhibiting picornavirus genomic features, isolated from breeding duck farms experiencing reduced egg production. Both strains possess the distinct feature of seven predicted 2A polypeptides. This, in conjunction with analyses of genome structure and homology, offers a foundation for exploring the potential taxonomic affiliation of these viruses with the genus *Aalivirus*.

## Materials and methods

### Collection of samples and isolation of virus

The two cases under investigation originated from breeding duck farms located in Nanchang, Jiangxi, in February 2022, and Peixian, Jiangsu, in March 2023, respectively. Both farms reported a marked decline in egg production among the breeding ducks. Specifically, the affected ducks in Nanchang were 30-week-old Mallards, which experienced a 30% reduction in egg production within 1 month, with the incidence of deformed eggs reaching 20%. In contrast, the affected ducks in Peixian were 28-week-old Pekin ducks, whose egg production rate decreased by approximately 45% within a span of 10 days. Tissue samples from the liver, spleen, and oviduct of the death ducks were collected and homogenized with PBS buffer at pH 7.4 in a 1:3 ratio. The homogenate underwent three cycles of freezing and thawing at −80 °C, followed by centrifugation at 12,000 × *g* for 20 min. The resulting supernatant was filtered through a 0.22 μm filter (Millipore, USA) and inoculated into 10 day old specific pathogen-free (SPF) chicken embryos with serial passaging conducted five times.

### Preparation of viral nucleic acid and metagenomic analysis

The cultured viruses were subjected to centrifugation at 12,000 × *g* and 4 °C for 30 min to obtain supernatant for viral RNA extraction using an Omega Viral DNA/RNA Kit (Omega, GA, USA). A sequencing library was constructed using the NEBNext Ultra II Directional RNA Library Prep Kit for Illumina (NEB, USA). Sequencing of the library and subsequent bioinformatics analysis were carried out as previously described (7). Briefly, the library was subsequently sequenced on the Illumina NovaSeq 6000 (PE150) sequencing platform (USA). Sequencing reads were first quality controlled using fastp (v0.20.0) (8). The remaining quality reads were assembled *de novo* using SPAdes (v3.13.0). The assembled contigs of viruses were confirmed by comparing against the NCBI non-redundant protein database (nr) using blastx.

### RT-PCR/PCR detection of the duck susceptible viruses

The RT-PCR or PCR were used to detect the virus using a set of primers designed in our own lab (Table 1) for duck susceptible viruses including avian influenza virus (AIV), duck enteritis virus (DEV), DHAV, duck parvovirus (DPV), duck reovirus (DRV), duck Tembusu virus (DTMUV) and duck picornavirus (PIV) FC22 strain. The PCR was performed in 25 μL reaction mixtures including 12.5 μL 2 × Taq PCR StarMix, 0.5 μL each primer (20 μmol/L), 2.0 μL DNA template, and 9.5 μL of water. Cycle conditions: one cycle at 95 °C for 2 min; and then 35 cycles at 95 °C for 20 s, 52 °C for 20 s, and 72 °C for 30 s; and a final extension step of 10 min at 72 °C. The RT-PCR was performed in 20 μL reaction mixtures including 10.0 μL 2 × One Step Mix, 0.5 μL each primer (20 μmol/L), One Step Enzyme Mix 1 μL, 2.0 μL RNA template, and 6.0 μL of water. Cycle conditions: 50 °C for 20 min; one cycle at 95 °C for 2 min; and then 35 cycles at 95 °C for 20 s, 52 °C for 20 s, and 72 °C for 30 s; and a final extension step of 5 min at 72 °C.

**Table 1:** Primers used in duck susceptible viruses detection.

Primers	Sequences (5′–3′)	Position^a^	Fragment size (bp)
AIV-F	TTCTAACCGAGGTCGAAAC	21	229
AIV-R	AATCGTCTACGCTGCAGTCC	249	
DEV-F	TCGCCTGCCAACTTAT	106,417	700
DEV-R	TCCTGGAACAATCACAAC	107,106	
DHAV1-F	GACTGTGCAACACGCTTCAAC	2,234	473
DHAV1-R	AATCTACTTCATCCCCAGACTG	2,706	
DHAV3-F	TGTGTATCCTATGAGCAGGCCA	6,320	646
DHAV3-R	AGCCCAACACAGCAAGCAC	6,965	
DPV-F	GCAATTACCAGTGGAACCTCTC	3,175	212
DPV-R	GTCTGATCCTGCGTTGTGACT	3,386	
DRV-F	TCATTCATTTGGGCAGCGG	1,206	226
DRV-R	AGTAGTGTGTAAAGCATGGACT	1,431	
DTMUV-F	ATGTCTAACAAAAAACCAGG	96	363
DTMUV-R	CAGCCCAGCAACTATCG	458	
PIV-F	GGAAGTTAGAGGTCTGGGTA	523	414
PIV-R	TGATGGATTGTAGGCTTGT	936	

a Position correspond to: AIV (MN209318), DEV (JF999965), DHAV1 (NC_008250), DHAV3 (KU860089), DPV (KY511124), DRV (MK749407), DTMUV (MH414568), PIV (MN102111).

### Genome characterization and phylogenic analysis

Sequences of 44 representative members of different picornavirus genera were downloaded from GenBank. Multiple sequence alignments were performed using Clustal Omega.^1^ Similarity calculations were performed by using GeneDoc software ver. 2.7. The secondary structures of the 5′ UTR (untranslated region) and 3′ UTR were predicted by the Mfold program,^2^ visualized using the Structure Editor software ver. 6.5. The phylogenetic trees based on the aa sequence were constructed using the neighbour-joining method with the Poisson correction method of MEGA software ver. 11.0 (9).

### Farm sample testing

Based on the genome sequences of NC0246, PX0394, and the published genome sequence of duck aalivirus A1 and DERSV, a conserved region was selected for the design of detection primers. The upstream primer was designated as DALV F: 5′-TTTCAACGKCTGGCCCAC-3′, lies at 40–57 base pairs (bp) in NC0246 strain and 38–55 bp in PX0394 strain, and the downstream primer as DALV R: 5′-CAGGCAGTYCCCCTTATCAG-3′, lies at 456–475 bp in NC0246 strain and 424–443 bp in PX0394 strain. The amplified fragment length was 406 bp, and the annealing temperature was set at 55 °C. A total of 144 cloacal swabs were collected from healthy breeding ducks across eight farms located in six regions of Shandong Province, Anhui Province, and Hebei Province of China (Supplementary Table S1). These samples were processed according to established methods and subsequently analyzed using the aforementioned primers. Positive samples were sequenced to confirm the presence of related viral infections.

## Results

### Virus isolation

Disease samples from Nanchang, Jiangxi Province, and Peixian, Jiangsu Province, were inoculated into SPF chicken embryos. All the infected chicken embryos died between 72 and 120 hpi (hours postinfection) showing hyperemia, hemorrhage, and edema. The viral nucleic acid were negative for AIV, DEV, DHAV, DPV, DRV, DTMUV and PIV using RT-PCR or PCR method. This process resulted in the isolation of one virus from each location, designated as Duck/NC0246/China/2022 (abbreviated as NC0246) and Duck/PX0394/China/2023 (abbreviated as PX0394), respectively.

### Genome sequencing

**Figure 1:**
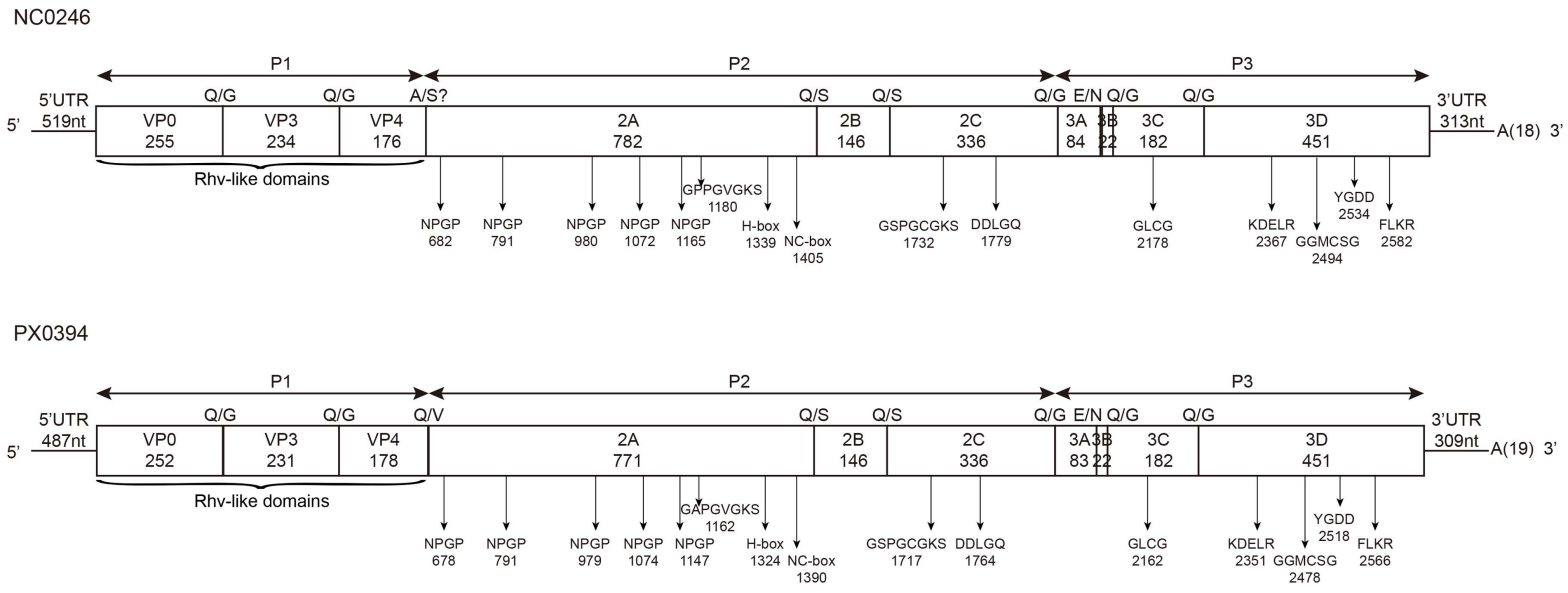
Predicted genome organization, conserved motifs, and the predicted cleavage sites of the duck-origin picornavirus NC0246 (GenBank accession no. PV583775) and PX0394 (GenBank accession no. PV611531). aa lengths were indicated in each gene box. The positions of the conserved picornavirus aa motifs were indicated with the first aa positions. Predicted potential N-terminal cleavage sites along the coding region were indicated above each gene junction in italics.

The complete genome sequences of NC0246 (GenBank No. PV583775) and PX0394 (GenBank No. PV611531) were determined through genome sequencing. For PX0394, sequencing on the Illumina NovaSeq platform generated 26,891,916 raw reads, with an average GC content of 49.75%. Bioinformatic analysis identified 10,901 reads with significant similarity to viral proteins, of which 1,646 were classified within the family *Picornaviridae*. The NC0246 genome comprises 8,839 nucleotides (nt) with a G + C content of 42.62%, along with a polyadenylated tract measuring 18 nt. This genome includes a 5′-untranslated region (5’-UTR) and a 3′-untranslated region (3’-UTR) of 519 and 313 nt, respectively, at each terminus. It contains an open reading frame (ORF) of 8,007 nt, encoding 2,668 aa residues, and is predicted to encode the P1 (665 aa), P2 (1,264 aa), and P3 (739 aa) regions. In contrast, the PX0394 genome is 8,755 nt long with a G + C content of 40.88%, and it includes a polyadenylated tract of 19 nt. The genome features a 5′-UTR and a 3′-UTR of 487 and 309 nt, respectively. Its ORF spans 7,959 nt, encoding 2,652 aa, and is predicted to encode the P1 (661 aa), P2 (1,253 aa), and P3 (738 aa) regions (Figure 1).

### Analysis of coding regions

The P1 regions of the genomes of NC0246 and PX0394 encode three capsid proteins: VP0, VP3, and VP1. In NC0246, the lengths of VP0, VP3, and VP4 are 255 aa, 234 aa, and 176 aa, respectively, while in PX0394, the lengths are 252 aa, 231 aa, and 178 aa, respectively. Notably, no cleavage sites for the formation of VP4 and VP2 were identified in the VP0 protein of either species, a characteristic consistent with other members of the *Avihepatovirus* (4, 6). Homology analysis revealed that the P1 region of NC0246 was closely related to duck aalivirus A1, PX0394, and DERSV, with aa identities of 37.37, 36.32, and 35.18%, respectively. Conversely, the P1 region of PX0394 was closely related to DERSV, DHAV-3, and DHAV-1, with aa identities of 64.44, 37.28, and 37.16%, respectively (Table 2).

**Table 2:** Comparisons of aa sequence similarity of NC0246 and PX0394 with the closely related picornaviruses based on the P1, 2C, 3D, 2B and 3A-3B regions, respectively.

Picornaviruses	NC0246					PX0394				
	P1	2C	3D	2B	3A-3B	P1	2C	3D	2B	3A-3B
NC0246	100	100	100	100	100	36.32	83.63	69.62	71.23	75.00
PX0394	36.32	83.63	69.62	71.23	75.00	100	100	100	100	100
Chicken Orivirus A-KM203656	27.26	38.69	39.86	/	/	30.45	38.99	41.29	/	/
Quail Picornavirus-NC016403	27.91	28.85	28.70	/	/	27.56	29.43	29.59	/	/
Duck Anativirus A1-NC006553	25.23	46.10	30.07	/	/	19.80	31.48	29.30	/	/
Avian Encephalomyelitis Virus-NC003990	22.68	28.25	27.07	/	/	19.45	29.37	29.43	/	/
Chicken Picornavirus-NC024765	32.28	27.05	26.71	/	/	19.05	26.80	27.06	/	/
Chicken Proventriculitis Virus-KJ690629	21.19	33.33	27.86	/	/	20.71	32.16	29.10	/	/
Duck Egg-reducing Syndrome Virus-OL956952	35.18	75.30	68.74	55.33	72.12	64.44	76.49	73.61	56.67	76.19
Duck Hepatitis A Virus 3-KU860089	33.04	50.17	49.04	46.60	29.87	37.28	51.88	50.48	46.15	32.43
Duck Hepatitis A Virus 1-NC008250	33.93	48.66	49.04	43.69	29.25	37.16	49.41	50.71	45.19	37.33
Duck Picornavirus-MN102111	31.98	43.79	44.55	/	/	32.12	44.08	45.60	/	/
Duck Aalivirus A1-NC023985	37.37	58.51	61.54	47.37	48.00	35.31	59.40	62.30	56.95	46.08
Turkey Megrivirus-HM751199	22.95	32.94	27.86	/	/	/	32.16	29.10	/	/
Duck Megrivirus-NC024120	23.29	33.33	25.62	/	/	22.22	33.73	26.80	/	/

The aa sequence identity is shown as a percentage. /: No significant similarity found.

The P2 region of the NC0246 and PX0394 genomes encodes three non-structural proteins: 2A, 2B, and 2C. The 2A proteins of NC0246 and PX0394 are composed of 782 aa and 771 aa, respectively. The 2B protein is 146 aa in length for both genomes, while the 2C protein is 336 aa long, each containing conserved sequences such as GSPGCGKS and DDLGQ. Notably, both NC0246 and PX0394 possess five predicted NPGP sites and a potential cleavage site within the 2A protein, segmenting it into five NPGP-containing regions (2A1-2A5), an AIG1-like region (2A6), and a Parechovirus-like region (2A7) (Figure 1). The aa sequence analysis reveals that the 2A protein of PX0394 exhibits the highest similarity with the published MW24 sequence (GenBank No. MH453803) (10), whereas the 2A protein of NC0246 closely resembles the DERSV structure (data not shown) (6). The primary distinction between the two strains lies in the fact that NC0246 lacks 73 aa in the 2A4-2A5 region compared withPX0394 (Supplementary Figure S1). The 2C proteins of both PX0394 and NC0246 exhibit two conserved motifs, GSPGCGKS and DDLGQ, which are implicated in the ATPase activity of the proteins (Figure 1). Notably, the 2C protein of NC0246 was closely related to PX0394, DERSV, and duck aalivirus A1, with aa identities of 83.63, 75.30, and 58.51%, respectively. Similarly, the 2C protein of PX0394 shows similarity values of 83.63, 76.49, and 59.40% with NC0246, DERSV, and duck aalivirus A1, respectively. Furthermore, the 2B protein of NC0246 exhibits similarity of 55.33 and 47.37% with DERSV and duck aalivirus A1, respectively. In comparison, the 2B protein of PX0394 displays similarity values of 56.67 and 56.95% with DERSV and duck aalivirus A1, respectively, and 45.19 and 46.15% with DHAV-1 (NC008250) and DHAV-3 (KU860089), respectively (Table 2).

The P3 region of NC0246 and PX0394 encompasses four nonstructural proteins: 3A, 3B^VPg^, 3C^pro^, and 3D^pol^. The 3A protein comprises 84 aa in NC0246 and 83 aa in PX0394. The 3B^VPg^, 3C^pro^, and 3D^pol^ proteins in both sequences consist of 22 aa, 182 aa, and 451 aa, respectively. All these proteins contain the GLCG motif, as well as the KDELR, GGMCSG, YGDD, and FLKR motifs, indicating a high degree of conservation in this region. The 3D^pol^ protein of NC0246 was closely related to PX0394, DERSV, and duck aalivirus A1, with aa identities of 69.62, 68.74, and 61.54%, respectively. The similarity percentages of 3D^pol^ protein for PX0394 with NC0246, DERSV, and duck aalivirus A1 are 69.62, 73.61, and 62.30%, respectively (Table 2). Based on BLAST analysis, the 3A-3B protein of NC0246 shows 72.12 and 48.00% similarity with DERSV and duck aalivirus A1, and 29.25 and 29.87% similarity with DHAV-1 (NC008250) and DHAV-3 (KU860089), respectively. Similarly, the 3A-3B protein of PX0394 shows 76.19 and 46.08% similarity with DERSV and duck aalivirus A1, respectively, and 37.33 and 32.43% similarity with DHAV-1 (NC008250) and DHAV-3 (KU860089) (Table 2).

### 5′- and 3′-UTR analysis

**Figure 2:**
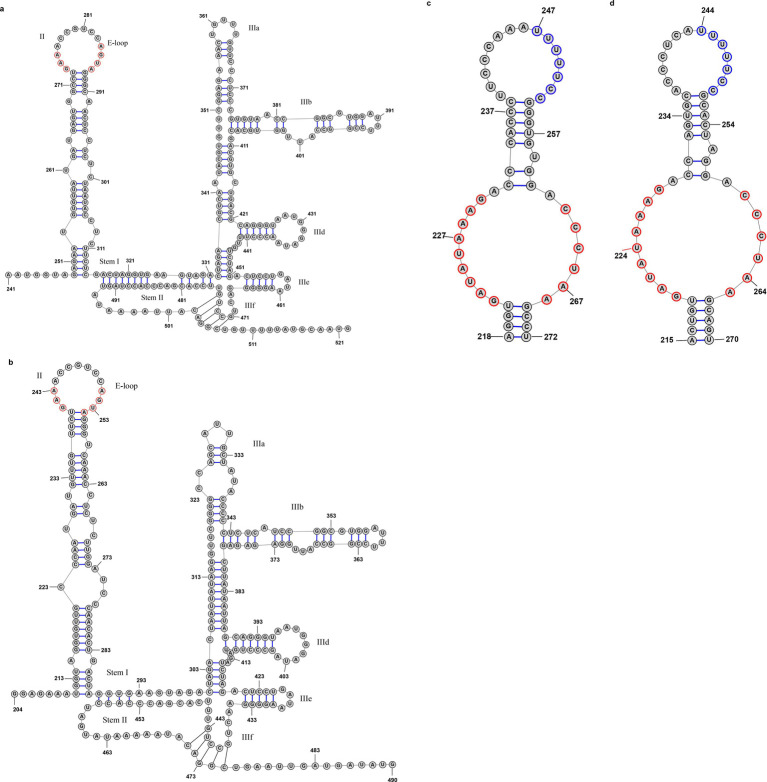
Predicted secondary 5′-and 3′-UTR structure of duck-origin picornavirus NC0246 and PX0394. These structures of 5′-UTR include conserved E-loop configurations with GAA and AGUA motifs (red-marking) within domain II, as well as elements IIIa, IIIb, IIId, IIIe, and IIIf within domain III containing the conserved motifs AUUU, AAUGGGAUA, GAUA, and CUGCCU, respectively. Secondary structure of 3’-UTR adopts a ‘barbell-like’ conformation including a conserved nucleotide sequence of 9 + 6 nt and a 6 nt polypyrimidine tract (red-marking). **(a)** 5′-UTR of NC0246, **(b)** 5′-UTR of PX0394, **(c)** 3′-UTR of NC0246, **(d)** 3′-UTR of PX0394.

The predicted lengths of the 5′-UTR for NC0246 and PX0394 are 519 nt and 487 nt, respectively. Both exhibit secondary structures characteristic of type IV-like internal ribosome entry sites (IRESs), akin to those observed in previously documented members of the genus *Avihepatovirus*. Specifically, domain II features a conserved E-loop structure with GAA and AGUA motifs, while domain III comprises elements IIIa, IIIb, IIId, IIIe, and IIIf, with IIIb, IIId, IIIe, and IIIf containing the conserved motifs AUUU, AAUGGGAUA, GAUA, and CUGCCU, respectively (Figures 2a,b). Despite their structural similarities, the domain II of NC0246 is 12 nt shorter than that of PX0394, resulting in a comparatively reduced length for NC0246. Furthermore, neither NC0246 nor PX0394 exhibits the characteristic ‘8-like’ structure in domain I of type IV IRESs, a feature present in several other members of the genus *Avihepatovirus* and avian picornaviruses.

The 3′-UTR of NC0246 measures 313 nt in length, accompanied by a poly(A) tail of 18 nt. In contrast, PX0394 exhibits a 3′-UTR length of 309 nt with a poly(A) tail of 19 nt (Figure 1). Secondary structure analysis has demonstrated that the 3′-UTR of both sequences adopts a ‘barbell-like’ conformation (Figures 2c,d). This structure includes a conserved nucleotide sequence of 9 + 6 nt and a 6 nt polypyrimidine tract, which aligns with the structural characteristics observed in other duck-derived picornaviruses, such as duck aalivirus A1 (3).

### Phylogenetic tree analysis

**Figure 3:**
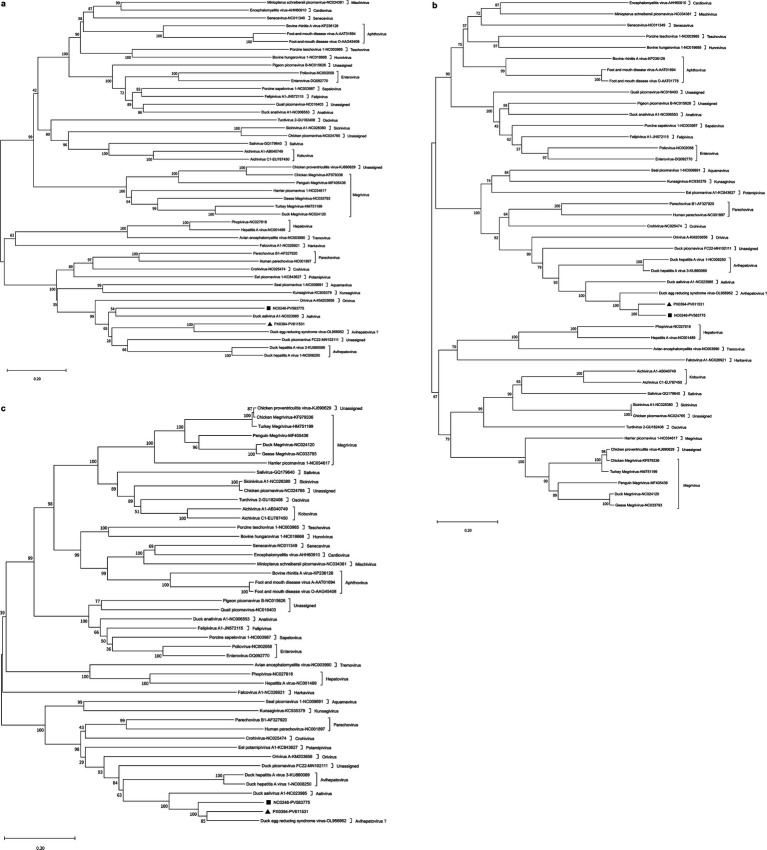
Phylogenetic relationship of duck-origin picornavirus NC0246 and PX0394 with other picornaviruses. The phylogenetic trees based on the aa sequences of P1 **(a)**, 2C **(b)** and 3D **(c)** were constructed using the neighbour-joining method and 1,000 bootstrap replications. The percentage of replicate trees in which the associated taxa clustered together in the bootstrap test were shown next to the branches. The scale bar indicates the number of substitutions per site. The NC0246 and PX0394 identified in this study were marked with black triangle and black square, respectively.

The aa sequences of the P1, 2C, and 3D proteins from NC0246 and PX0394 were subjected to homology analysis alongside 44 other members of the picornavirus family, resulting in the construction of an evolutionary tree. This analysis revealed that, for the P1 protein, NC0246 clustered within the same branch as duck aalivirus A1, while PX0394 grouped with DERSV (Figure 3a). In the case of the 2C proteins, NC0246 and PX0394 were found to be in the same branch, with both of them being closest to DERSV and duck aalivirus A1 (Figure 3b). For the 3D proteins, PX0394 exhibited the closest relationship to DERSV, followed by NC0246 and duck aalivirus A1, respectively (Figure 3c). Overall, the findings suggest that NC0246 and PX0394 are both closely related to DERSV and duck aalivirus A1 across the P1, 2C, and 3D proteins, with additional proximity to duck hepatitis A virus and duck picornavirus FC22.

### Farm sample testing

A total of 144 cloacal swabs collected from healthy breeding ducks were detected, and two out of six cloacal swabs from one farm in Qingdao, Shandong Province were positive (Supplementary Table S1). Then, the PCR products from the positive samples were sequenced and analyzed. The results showed that the sequences shared the closest relationship to PX0394 (data not shown).

## Discussion

In recent years, numerous duck-derived picornaviruses have been identified and documented, predominantly classified under the genera *Aalivirus*, *Anativirus*, *Avihepatovirus*, *Sicinivirus*, and *Megrivirus*, although a few pathogens remain with an unresolved taxonomic classification (3, 5, 6, 11–13). To date, with the exception of the AH15 strain, which features a predicted L protein (12), all duck-derived picornavirus genomes exhibit the structural organization of 5′UTR-P1 (VP0-VP3-VP1)-P2 (2A-2B-2C)-P3 (3A-3B-3C-3D)-3′UTR-poly(A). The genomes of NC0246 and PX0394, as reported in this study, also display the same structural characteristics. The P1 region is anticipated to encode three structural proteins: VP0, VP1, and VP3. Notably, no predictable cleavage site for VP0 has been identified, a feature consistent with viral members of the *Aalivirus*, *Avihepatovirus*, and *Megrivirus* genera (3–5). The P2 region encodes three non-structural proteins 2A, 2B, and 2C that are implicated in viral assembly and virulence. Among these, the 2A protein is particularly notable. Both NC0246 and PX0394 exhibit extended 2A sequences, and bioinformatics predictions indicate that each possesses seven distinct 2A polypeptides. Notably, polypeptides 2A1 through 2A5 contain ribosome skipping sites characterized by the NPGP sequence, while 2A6 features the GxxGxGKS motif typical of P-loop NTPases, and 2A7 includes the Hbox/NC motif. However, the structural composition of the 2A polypeptides in NC0246 and PX0394 is not identical. Specifically, NC0246 exhibits a deletion of 73 aa in the region corresponding to 2A4-2A5 when compared to PX0394. Furthermore, an analysis of the overall aa homology of the 2A polypeptides revealed that the 2A protein of PX0394 was most similar to the published MW24, whereas the 2A protein of NC0246 closely resembled the published structure of DERSV. This suggests that while the structural configurations of the two proteins are similar, their evolutionary origins differ. The 73-aa deletion in NC0246 compared to PX0394 may be associated with cross-species transmission and adaptation to new environments, as the 2A region of MW24 (identified in wild birds in Australia) was highly similar to that of PX0394, whereas the 2A region of DERSV (isolated from duck farms in China) was highly similar to that of NC0246 (6, 9). Therefore, future research should focus on utilizing reverse genetics systems to generate recombinant viruses with specific 2A mutations and assess their phenotypic changes in both *in vitro* and *in vivo* models. Additionally, a review of the existing literature indicates that picornaviruses with multiple tandem 2A polypeptides predominantly originate from avian species, implying that this structural characteristic may be associated with infection mechanisms adapted to avian hosts (14). The 2A protein of picornaviruses serves as a key factor in viral polyprotein processing, host modulation, and viral replication (15). However, picornaviral 2A proteins have evolved diverse, genus-specific functions. These include protease activity, involvement in host translation shutoff, modulation of stress granule dynamics, and even roles in viral RNA replication (16, 17). Recently, little is known about the geographic distribution, host spectrum or pathogenic potential, and the diversity of picornaviruses circulating among birds, although a number of known avian picornavirus species with complete genome was identified. Unlike many mammalian picornaviruses that typically possess a single 2A gene, avian viruses (especially those from ducks) often feature a unique genome organization with multiple, tandem 2A genes. Meanwhile, the precise functions of each individual 2A protein in tandem arrays are not yet understood, except for DHAV. For instance, the genome of DHAV-1 encodes three tandem 2A proteins (2A1, 2A2, and 2A3). Research shows that DHAV-1 2A1 contains a conserved ‘-GxExNPGP-’ motif at its C-terminus, which mediates a ‘ribosome skip’ during translation, enabling the co-translational ‘cleavage’ of polyproteins from a single open reading frame (18). DHAV-1 2A2 can induce apoptosis in primary cell culture and inhibit interferon beta production (19, 20). In this study, the seven tandem 2A proteins were identified, which might imply that the more complex mechanism of viral infection in avian hosts. This unique genetic architecture may enhances the virus’s ability to modulate the host cellular environment, promote its own replication, and drive pathogenesis (21). However, the function of the seven tandem 2A proteins in virus replication need to be explored further.

The 5’ UTR of avian picornavirus predominantly exhibits a Type IV IRES, a structure that facilitates viral protein synthesis via cap-independent translation and necessitates host factors, such as eIF4G and PTB, for the binding of specific structural domains (14, 22). The 5’ UTRs of both NC0246 and PX0394 encompass complete domain II, characterized by GAA/AGUG motifs, and domain III, which includes substructures IIIa to IIIf, aligning with the features of Type IV IRES found in *Avihepatovirus*. Notably, the domain II of NC0246 is 12 nucleotides shorter than that of PX0394, potentially leading to a compression of the stem-loop structure. This compression may result in a fine-tuning of the E-loop spatial conformation, thereby influencing the binding efficiency of host factors. However, domain I of the 5’ UTRs in both NC0246 and PX0394 lacks the ‘8-like’ structure commonly observed in other Type IV IRESs (14), indicating the possible emergence of a novel IRES subclass.

The ‘Barbell-like’ structure is observed in the 3′UTR of both NC0246 and PX0394, characterized by the original 9 + 6 motif: 5′-GAUAUAAAG-3′ + 5′-CCCUAA-3′. This motif is highly conserved among avian picornaviruses and is identical to those found in duck hepatitis A virus-1 (DQ219396), duck hepatitis A virus-3 (KP995438), duck/FC22/China/2017 (MN102111), duck Aalivirus A GL/12 (KJ000696), and turkey/M176/2011/HUN (JQ691613) (11, 23). Additionally, both species exhibit a 7-nucleotide poly(Y) tract within the ‘Barbell-like’ structure, although its length is shorter than that observed in most avian-derived viruses. This ‘Barbell-like’ structure enhances the efficiency of type IV IRES through long-distance interactions with the 5’ UTR, where the poly(Y) tract serves as a polypyrimidine tract-binding (PTB) protein binding site to facilitate ribosome assembly (22). Beyond avian picornaviruses, similar ‘Barbell-like’ structures have been identified in the 3’ UTR of certain mammalian picornaviruses and caliciviruses (14, 24–26), suggesting analogous functional mechanisms and evolutionary origins.

Based on the results of homology and phylogenetic tree analyses, NC0246 and PX0394 exhibit the closest genetic relationship to DERSV and duck aalivirus A1. Their 2C and 3D proteins demonstrate over 58% similarity with DERSV and duck aalivirus A1, which is higher than their similarity with other viruses. However, substantial divergence is observed in the P1 region: the P1 protein of NC0246 shows 35.18% similarity with DERSV and 37.37% with duck aalivirus A1, whereas the P1 protein of PX0394 exhibits 64.44% similarity with DERSV and 35.31% with duck aalivirus A1 (Table 2). This suggests greater variability in the P1 proteins between NC0246 and PX0394. Phylogenetic analysis further indicates that NC0246 and PX0394 form the nearest branches to DERSV and duck aalivirus A1, while DHAV-1 and DHAV-3, which display secondary homology, are positioned on a distinct branch. Consequently, it is inferred that NC0246 and PX0394 are most closely related to DERSV and duck aalivirus A1. According to the current genus classification criteria,^3^ the following conditions must be met: i. Members of different genera exhibit distinct genomic structural characteristics; ii. There should be greater than 66% similarity differences in the P1 protein, with similarity differences in the 2C and 3D proteins exceeding 64%; iii. Members of different genera should lack homology in the L, 2B, 3A, 3B, and other proteins. Based on the results, it was observed that i. NC0246, PX0394, DERSV, and duck aalivirus A1 share similar 5′UTR IRES, 3′UTR ‘barbell-like’ structures, and 2A proteins characterized by multiple NPGP-containing regions, an AIG1-like region, and a parechovirus-like region arranged in tandem; ii. The similarity of the P1 proteins of NC0246 and PX0394 with those of DERSV and duck aalivirus A1 exceeds 35%, while the similarity of the 2C and 3D proteins with DERSV and duck aalivirus A1 is greater than 58%; iii. While the proteins 2B, 3A, 3B of NC0246 and PX0394 exhibit some degree of similarity with DHAV, their similarity with duck aalivirus A1 and DERSV is markedly greater. Based on the preceding analysis, the genomic architecture and gene homology of NC0246 and PX0394 indicate a close phylogenetic relationship with duck aalivirus A1, with DERSV also sharing this close evolutionary association. These results suggest a potential affiliation of NC0246, PX0394 and DERSV with the genus *Aalivirus*, though definitive classification awaits further validation in accordance with strict International Committee on Taxonomy of Viruses (ICTV) taxonomic criteria.

Picornaviruses identified as pathogenic to avian species include avian encephalomyelitis virus (AEV) (27), turkey hepatitis virus (THV) (28), DHAV (29), and DERSV (6), among others. Additionally, certain avian-origin gallivirus, megrivirus, and sapelovirus have been associated with subclinical manifestations such as stunting andenteritis syndrome, malabsorption syndrome, and growth depression; however, the pathogenicity of most avian-origin picornaviruses remains largely unexplored (4, 30, 31). Notably, NC0246 and PX0394 were isolated from breeding ducks that exhibited decreased egg production. DERSV, which has been previously illustrated to share a close evolutionary relationship with these isolates, was also obtained from ducks experiencing reduced egg production. This correlation suggests that NC0246 and PX0394 may be associated with the observed decline in egg production among breeding ducks. Liao et al. (5) identified the presence of duck megrivirus in duck flocks experiencing reduced egg production, while Zhang et al. (21) demonstrated that DHAV-1 can similarly lead to decreased egg production in adult ducks. Infection by avian picornaviruses, such as DHAV-1 and DERSV, leads to a decline in egg production through multifactorial and synergistic mechanisms. The key pathology may involves direct damage to the reproductive system showing ovarian hyperemia and hemorrhage, necrosis, atrophy and distortion, and then subsequent systemic physiological disruptions (6, 21). These findings suggest that various duck-associated picornaviruses may contribute to symptoms of reduced egg production. Analysis of cloacal swab samples revealed that only 2 out of 144 samples, collected from eight farms across six regions, tested positive for the virus, indicating its low prevalence in farm settings. Notably, the positive samples were obtained from healthy ducks rather than those with reduced egg production, suggesting that the virus may be a conditionally pathogenic agent, becoming pathogenic only under specific stress conditions that compromise the duck’s immune systems. Nevertheless, the pathogenicity of pathogens is associated with various factors, including the biological characteristics of the strains, the infectious dose, host type, age, and other variables (32–34). Further investigation is required to elucidate these relationships through the identification of viral characteristics and animal experiments.

## Data Availability

The original contributions presented in the study are publicly available. These data can be found in GenBank, accession numbers PV583775 and PV611531.

## Footnotes

1
https://www.ebi.ac.uk/jdispatcher/msa/clustalo

2
https://www.unafold.org/mfold/applications/rna-folding-form.php

3
https://ictv.global/report/chapter/*picornaviridae*/*picornaviridae*

## Supplementary material

SUPPLEMENTARY FIGURE S1 Comparison of putative 2A peptides of NC0246 and PX0394 with other picornaviruses. The NPGP sites and a potential cleavage site within the 2A protein were marked with black triangle. The dot represents the absence of aa. The conserved motifs were marked with red in the box.

SUPPLEMENTARY TABLE S1 The results of diagnostic RT-PCR for 144 samples.
