# Supplementary material for: Genomic characterization of two duck-origin picornaviruses with seven putative 2A peptides
Source: Front Vet Sci. 2026 Apr 23;13:1753959. doi: 10.3389/fvets.2026.1753959 (PMC13149134; doi:10.3389/fvets.2026.1753959)
Supplement: SUPPLEMENTARY FIGURE S1 — Comparison of putative 2A peptides of NC0246 and PX0394 with other picornaviruses. The NPGP sites and a potential cleavage site within the 2A protein were marked with black triangle. The dot represents the absence of aa. The conserved motifs were marked with red in the box. [file Image_1.pdf]

```
                         1        10        20        30        40        50
GL12-NC023985            GATNSSLLKLAGDVEENPGPLQIEGKPYRV.IQKGPNRREIWSTFTHNNLKVKSRVIET.
DHAV-NC008250            ............................................................
cftwhg06-MT138343        LGGWIQDLTECGDVESNPGPNFENRLLEFKGFYFA.VSSNTTHGFLS.....KTNVR...
Abbotsbury2016-MW588066  ATAFIRDLTCDGDVESNPGPTLVCRV.DQVEDGFEFNMIQKWKGFIT.....KNRVSYHS
NC0246                   SRMWVRDLTADGDVESNPGPATFRIIELKNPDAYVTYECREWRGLLV.....ENKVLHWT
DESV-OL956952            ATSYVKDLTIDGDVESNPGPQYVRQRIDL.GEEYIQYEFKKWRGLLV.....TNKVLVWS
PX0394                   VTNFIRDLTIDGDVESNPGPVFEQKRIDLPGLGYVWMDHRKWKGVMI.....SNEVLYWS
MW24-MH453803            VSEYLKDLTIDGDVESNPGPVFEQRKIDLPGLGYVWMDHRSWKGFLI.....KNEVLYWS
                                            ▲

                         60        70        80        90        100       110
GL12-NC023985            KGTLRIRRTTVQTCFNTRDLSCKPVSVMFRIENETPFGFDCQTMYDFITEQHRYVHYTIK
DHAV-NC008250            ............................................................
cftwhg06-MT138343        ..........HLTCYTDGDQF......IFSRHTVEVNNLLTKYREDIFSTQAGSFSLIRK
Abbotsbury2016-MW588066  WHLQRPPSTPVFSRFSHRRSI......LWTYQEVEVANWSK......SNGQDISITYITK
NC0246                   YHPGPIPTPCSISCHEVVRKY......LVKKVVEAEYDLET.......PTGPRRIYHVIE
DESV-OL956952            LHKGPYPSPASFTCYEQTKQT......LWKKKMFIEWELDY.......NGVTYWSRQEIE
PX0394                   FHDGEFPTPATISCHEAKRKC......LWKSKIIISLEFYH.......DNKTYWQKIIKT
MW24-MH453803            YHEGGFPSPAVISCHEAKRKF......MWKSKLIVNLEFYH.......GQKTYWQKTIKN

                          120       130       140       150                 160
GL12-NC023985            KPFREPVTTSFEMPYDDPEWDRLLQAGDIEQNPGPDRVEEM.........RPYGKKITFS
DHAV-NC008250            ............................................................
cftwhg06-MT138343        KDWLGR....RFSKIIERSHIKDLTTEGIEPNPGPATYTVERNTLTTRQIRSYDEDEEFY
Abbotsbury2016-MW588066  RNFLGL....TKSTVRSSEWERDLTTEGIEPNPGPIVLQQTRYKNWSVQTRHFGAWNNYN
NC0246                   ....KK....LFRKPIERMFVKDLTMEGVEPNPGPANFSYAEINDHTVRSKTYGENMTHY
DESV-OL956952            RKWNFQ....KTCREVEGSYVKDLTEEGIEPNPGPADFSSFSSGFSSVTSRAYGENFTHY
PX0394                   RNWLGR....TQVRKLEGDFVKDLTTEGIEPNPGPSDFTYAEILNQTIRSRSYGESPSHY
MW24-MH453803            RDWLGR....LKVKKLEGDYVRDLTQEGVEPNPGPSNFTYSEINNQTIRSRSYGESDSHY
                                                           ▲

                         170       180       190       200       210       220
GL12-NC023985            QRQGRFLAKHYIRQYE.....FNVVKREDILLTYYDVEGYWPETPEATVHVYRTFLQHV.
DHAV-NC008250            ............................................................
cftwhg06-MT138343        WTQDTYSGHKYAAKVLRVHLKSNGIPSKPVFTIKNTVIRCLRGNKVKTVITFDT.ANGT.
Abbotsbury2016-MW588066  WTFDHFSNGSHHLNCIKIQQGG...HTIPFFLAKFETIASWDKTILIFKVGVR.VDNHWE
NC0246                   WETNYYTNGHHSLHCTTVHRKDQGIPSPAVFCAKVTTVASWFSVRVRVVVMIRSVHDDWK
DESV-OL956952            WQNNNYTNGYYTTNLTTVHAKSNGIPSPPVFTAKVTTTASWFTVKVRVVVSIR.THQGWK
PX0394                   WENNYYTNGNYSAHLTKVHLKANGIPSPPTFTVKVTVNVSWWSIKIRCVVMIR.IFGQWK
MW24-MH453803            WENNYYTNGSYSAHLTKIHLKANGVPSPPIFTVKITVNVSWWSIKIRCVIMMR.IFGQWK

                                             230                        240
GL12-NC023985            ......IIENF........LYE..LP....................VRPRGTKRF..RII
DHAV-NC008250            ............................................................
cftwhg06-MT138343        ............................................KVIK............
Abbotsbury2016-MW588066  FLRTKINPDKA.......VEFE..FDGNKDHFHFERYSTKNMSWTSIRPLSDRNVGVSMF
NC0246                   VFRTKFKISNWTFRNWKPIVFEENLPEIFDFHDLTTSNCPGSQWTVIKSKEDAHSAVSLS
DESV-OL956952            MFRAKFKLNRMTFARWNPIEFQETLPEIVAYSTVTNSGTRGSQWTVIRSTEVAGSAVSLS
PX0394                   ILRTKFKVKSRFFNQWSPIIFEENLPHERHHHQIFSSDSIGSQWVVIKSLGIGETAVSLC
MW24-MH453803            IFRTKFRVKSRFFNQWSPIVFEENLPGEQHHHDKFSSDVVGSQWVVIKSRGLGETAVSLS

                             250
GL12-NC023985            VERDGV......................................................
DHAV-NC008250            ............................................................
cftwhg06-MT138343        ........SVVPPGHET.......PLMWHEDILRCGDVEQNPGPRKWLTARHSYNLGEIR
Abbotsbury2016-MW588066  CDRNEVEILFYPAPQNQPHQFKFLKNSWRRDLTIDGDVESNPGPSQWLVHRSQGHDGELS
NC0246                   KTWDRVDVKIYPTELTNPINLKIADGGWVHDLTQDGDVESNPGPF.WDVDNYTVNFGDGT
DESV-OL956952            KTWDRIDVRVYPTQHTQPYLLKIGNAAWVRDLTEDGDVEENPGPTQWLCDRDMIHARDGW
PX0394                   KTWEKLEVKIYPVQGANPISIRVTDAG.................................
MW24-MH453803            KTWEKLEVKIYPVQGVNPISVRVTDAG.................................
                                                                  ▲

                                              260                 270       280
GL12-NC023985            .............EQTYMTGPYD............ALLQVYNALPTALKEIIPIPARPDP
DHAV-NC008250            ............................................................
cftwhg06-MT138343        VRMVIHILISEEHGVVQLVGPVIYDRNERAPGGKKRRIVEYYYDPNEEPYRKIYPCHEEC
Abbotsbury2016-MW588066  WKTETLVLIDIQGEDVFMVGPIINIKKTRRTGGAKKVVYTYYDGQSLEPVEHIEYCHRNC
NC0246                   MNVQTFVLMQKQGDDYHALGPRISVTKIRMRGGRKKVIYCWASNWTDRPTVLETKCHEEC
DESV-OL956952            MMVDTYILVKKNGDRMRMVGPLISVIKTKLNDGRSQVEYRWRRSLIHSPESVVYNCHESC
PX0394                   ............................................................
MW24-MH453803            ............................................................
```

```
                              290       300       310       320       330       340
GL12-NC023985            QWNNLQQAGDVEMNPGPEFRKADTQMTVDKKVTCFSTTVNKVTIQQEGFEVSKTVLLKKN
DHAV-NC008250            ............................................................
cftwhg06-MT138343        WVRDLTMDGDVESNPGPRKIV.......................................
Abbotsbury2016-MW588066  WLNDLTIDGDVESNPGPAFWRVDKSYGSDEF.................GSYTLTEFVQLF
NC0246                   WVHDPTQDGDVEPNPGPSATR....HECEKW.............TRDGVKYEKETWARVF
DESV-OL956952            WNRDLTIDGDVELNPGPRDPLPCSTVERKEW.............EHNGVLYYYTKYTKSF
PX0394                   WVRDLTIDGDVESNPGPRDPLPCTAWDRKEW.............ECNGVRYYYSKYTKSF
MW24-MH453803            WVRDLTVDGDVESNPGPSNPMHCTKMDSKEW.............EHNGIRYYYTKYTKSF

                              350       360              370         380       390
GL12-NC023985            TWFSRSHVETKVVYFKGHLSS.......MNVV...DSSWTPFETKETRVCWVPQ......
DHAV-NC008250            ............................................................
cftwhg06-MT138343        ............................................................
Abbotsbury2016-MW588066  NFDGHELCTGLIIKLQVHKKTNGAKRTTIFVTTN..........................
NC0246                   HYGGVNYAGGTVIEHVVMRKNNGARMDKFFVYKEDAGDCRSWVF................
DESV-OL956952            RWFGHVYEGNVSLVHVMEVHPDNRRKDTFK.LKDENGQIYDWVFKCHEKCWQKDPTQDGD
PX0394                   RWLGHVYEGDTSLIHIMEIHPQGKRKDTFQ.LRDRS.RVEKWVFKCHDRCWEEDLTQCGD
MW24-MH453803            RWFGHIYEGDTSLIHVMEIHPEGRRKDTFQ.LRDEN.RIEKWVFKCHDKCWVDDLTNCGD

                                       400                                      410
GL12-NC023985            ...AGEPYVTTVVSRSG..............................WFWRSE...HFNQ
DHAV-NC008250            ..........................................................SD
cftwhg06-MT138343        ............................................................
Abbotsbury2016-MW588066  ................................................MVDYDSVTTCCH
NC0246                   .........................................................KCH
DESV-OL956952            VEQNPGPYLEITTWRVGNVHITEHCYDGGLIIHQTFINWSNGAKKEVFIVEDRCYEFKCH
PX0394                   VEQNPGPYVIHNHWKVDNINVTEHIYNNGEIIHRVYYVNWSNGAKQEVFFFKDEMFIFKCH
MW24-MH453803            VEPNPGPYVIHNHWRVDNINITEHIYNGGEVVHRVYYVNWSNGAKQEVFYFRGEMFIFKCH

                                420       430       440       450       460       470
GL12-NC023985            TGGWVPDLTQCGDVESNPGPWDPERKHFCVVGPGGVGKSHVANLLTQTGMKLFKEACSPG
DHAV-NC008250            QIRNKKDLTT.EGVEPNPGP.......ILVVGKSGSGKSRLCNVLA..GVELFESKLSPR
cftwhg06-MT138343        .............................VVGPNQCGKTTLIRDAF..GVPVEPSKN...
Abbotsbury2016-MW588066  ERCWVRDLTRDGDIESNPGPWTPDDTVIVVLGPPGSGKSFVSNLIL..GSKVFFEQPDRN
NC0246                   ESCWDKDLTQDGDVEPNPGPWSPDTHSIAVLGPPGVGKSTIANLLI..GGPCFRTRTKYK
DESV-OL956952            EHCWVRDLTMDGDVEENPGPWSPDTKTIMVLGATGSGKSYAANKIL..GKEAFIHKLSTK
PX0394                   EGCWIEDITVDGDVESNPGPWTPDTRSIVVLGAPGVGKSTVANQLI..GAKCFRTRDQHK
MW24-MH453803            EGCWVEDLTVDGDVESNPGPWTPDTRSIVILGAPGAGKSTVANEVI..GAKCFRTRDQHN

                                480           490       500       510       520       530
GL12-NC023985            PVTKEQETR....EFAKRIITDTPCFSDIGQETKPKALDMYDCFVYVHQPGRFQKIEEKY
DHAV-NC008250            SVTMVHQVETVDIEGKKVTIVDS........PETPNYDGPISAFFYLIEAGRFTAEDKEY
cftwhg06-MT138343        .KKYSHDTY....NTAFGEIIEC........RQVPKLVDLVDCFILVVRPGLEGYR..EL
Abbotsbury2016-MW588066  KPSYCDSIR....THQNLIIVDS........GPFPNIKDFVSKFVFVENTLVSQTLTNQY
NC0246                   ESTYCNAER....VRGNLTIVDT........PTIPRFKDFVSSFIYVHRMGHHNVAEVEY
DESV-OL956952            SVTYCDQAI....THGNLTVIDT........APLPKLTDFVSGFVYVHKAGRFNAEEKAY
PX0394                   ESTYCMAER....THGQLTIYDT........PELPKIKDFVSGFIYVHRIGKFSVAEDVY
MW24-MH453803            ESTYCMSER....THGQLTVYDT........PELPKVKDFVSGFIYVHRIGKFNTAEEVY

                                    540       550       560       570       580
GL12-NC023985            EQELLKVFPNFWAHTVVVINFRGQEDEKAEQMQRYMEHTIYQKLFHKSQGRVLMIDNI..
DHAV-NC008250            IQMMRKRFPGFEKSTILILNRADELKTDDDIRNWVKTSGELDSLVRACGGRIAKFHRHKI
cftwhg06-MT138343        IVDMERSYPNWLSHSVIFFSQSPT...RQHPEKYLEKH.GFSSILFKCMGRVTDEPRVI.
Abbotsbury2016-MW588066  IDMLNRSYPNWQAHTVIYRNSTQS...............GSQDLEFQVMGRVAETRTQL.
NC0246                   VKALDREFPNWQAHAVLLLIQPDE...PIHPDTYMKETPFIHDLGFKMMGRITDYHEVA.
DESV-OL956952            LDLLDKMLPNWQAHAVLLVPQR..EVQKVYYVEDYIKNHKELSGLAFKMMGRITDSYEIA.
PX0394                   IKMLDREIPNWQAHCVLLLLQPEDEESKMNPDTYLKECQPIGNLAFQMMGRTTDYVEVA.
MW24-MH453803            IKMLDREIPNWQAHCVLLLKQPEEEDLKVNPDIYLKECQPIGNLAFQMMGRTTDYVEVA.

                               590       600       610       620       630       640
GL12-NC023985            ...DKIDELINFPPYHQHFAQLVYQNRGMYKHYGVKVGETVYHLNTSDIIESSLKGVARV
DHAV-NC008250            NRAKLLEKAATLPEFVSHLPRLVYKDRKMYRHYGVQCGSTVFHMDSENILESALNGEVTI
cftwhg06-MT138343        ...EGLLKAMGNGPYFSHLFKLVYKNRGAYRHYGIKNGSTVYHLNTGDILQSALEGTATV
Abbotsbury2016-MW588066  ...MDM.LDQLKSPYFHHLCKLVYKERQAYRHYGVRYQGKVFHLNTANILTSALQGKTDV
NC0246                   ...KKLCRECITSPYFYHMYQLVYKDRGAYRHYGVRARGATYHLSTEDILKSALSGTAKV
DESV-OL956952            ...KKMICECLPSPYFSHHYKLVYKNRGAYRHYGVLSHGRVFHLNTADILKSALSGSASV
PX0394                   ...KKMIIDCIHSPYFNHYYRLVYKDRGIYRHYGIKHRGAVYHMNTGNILKSSLSGTAQV
MW24-MH453803            ...KKMIVDCIHSPYFNHYYRLVYKDRGIYRHYGIKHKGAVYHMNTGNILKSSLSGTAQV
```

```
                            650       660       670       680       690       700
                              .         .         .         .         .         .
GL12-NC023985               QESEYSARWKPVDEVAYI.NGYNLFSSGAVEVEFNIDDNCDSWSRKMIGSNSPTQGARLK
DHAV-NC008250               KQEKWNGNWKPAGDHMQSTASLYLKSQTMPKFTFSIDENCETWARHLLGDYGETQGQIFK
cftwhg06-MT138343           HLDDDPQSWIESDETGYR.NAMHVVNAKLVNYKFSMDSNCETFARMFVDSDSPMQGDRIK
Abbotsbury2016-MW588066     QVDLDPTSWIETDEVSYT.NAISFVNSGAVSLDFNFDSNCETWAKLVMGNDTPCQSARLK
NC0246                      QIDHDPSGWIPAEPSEYR.NAAHLINAGAVELDFSFDDNCESWARSVLGDTGEHQGSRLK
DESV-OL956952               QVDHNPQEWIKAEENGYR.SALYLVNAGAIDLDFNFDSNCETWAKTILGSDQACQGHRLK
PX0394                      QLDYSTTGWIEAEPSEYR.SAYHLINAGAVEMEFNFDSNCESWARSVLGSTDEHQGARLK
MW24-MH453803               QLDYSTSGWIEAEPSEYR.SAYHLINSGAVEMEFNFDNNCESWARSVLGNTDEHQGARLK

                              710       720
                                .         .
GL12-NC023985               WCLSVAAAMAFISSMDMVSNE
DHAV-NC008250               ERLMWAAALGFFMTMKITTDQ
cftwhg06-MT138343           WGLTLAAASAFLFCSSSFSDQ
Abbotsbury2016-MW588066     WCLTVSAAMAFLYSGVYLENQ
NC0246                      WCLAITAAAGFLYSSLELEDQ
DESV-OL956952               WCLTLAAAAAFMFSGVHIEDQ
PX0394                      WCLTLAAAASFMFSGLVLENQ
MW24-MH453803               WCLTLAAAASFMFSGLVLEDQ
```
