# Supplementary material for: Genomic characterization of two duck-origin picornaviruses with seven putative 2A peptides
Source: Front Vet Sci. 2026 Apr 23;13:1753959. doi: 10.3389/fvets.2026.1753959 (PMC13149134; doi:10.3389/fvets.2026.1753959)
Supplement: SUPPLEMENTARY TABLE S1 — The results of diagnostic RT-PCR for 144 samples. [file Table_1.docx]

TABLE S1 The results of diagnostic RT-PCR for 144 samples

| Areas | Sample Type | Total No. | No. of Positive Samples |
| --- | --- | --- | --- |
| Suzhou, Anhui | cloacal swabs | 11 | 0 |
| Binzhou, Shandong | cloacal swabs | 11 | 0 |
| Qingdao, Shandong | cloacal swabs | 6 | 2 |
| Dongying, Shandong | cloacal swabs | 36 | 0 |
| Cangzhou, Hebei | cloacal swabs | 22 | 0 |
| Dezhou, Shandong | cloacal swabs | 58 | 0 |
| Total |  | 144 | 2 |
